# Smartphone Positioning and Accuracy Analysis Based on Real-Time Regional Ionospheric Correction Model

**DOI:** 10.3390/s21113879

**Published:** 2021-06-04

**Authors:** Qi Liu, Chengfa Gao, Zihan Peng, Ruicheng Zhang, Rui Shang

**Affiliations:** School of Transportation, Southeast University, Nanjing 211189, China; liuqi_0328@163.com (Q.L.); pzhseu@163.com (Z.P.); zrc_1996@seu.edu.cn (R.Z.); shangrui@seu.edu.cn (R.S.)

**Keywords:** GNSS, ionospheric delay, regional ionospheric model, Android smartphone

## Abstract

As one of the main errors that affects Global Navigation Satellite System (GNSS) positioning accuracy, ionospheric delay also affects the improvement of smartphone positioning accuracy. The current ionospheric error correction model used in smartphones has a certain time delay and low accuracy, which is difficult to meet the needs of real-time positioning of smartphones. This article proposes a method to use the real-time regional ionospheric model retrieved from the regional Continuously Operating Reference Stations (CORS) observation data to correct the GNSS positioning error of the smartphone. To verify the accuracy of the model, using the posterior grid as the standard, the electron content error of the regional ionospheric model is less than 5 Total Electron Content Unit (TECU), which is about 50% higher than the Klobuchar model, and to further evaluate the impact of the regional ionosphere model on the real-time positioning accuracy of smartphones, carrier-smoothing pseudorange and single-frequency Precise Point Positioning (PPP) tests were carried out. The results show that the real-time regional ionospheric model can significantly improve the positioning accuracy of smartphones, especially in the elevation direction. Compared with the Klobuchar model, the improvement effect is more than 34%, and the real-time regional ionospheric model also shortens the convergence time of the elevation direction to 1 min. (The convergence condition is that the range of continuous 20 s is less than 0.5 m).

## 1. Introduction

The rapid development of smartphones and low-cost GNSS chipsets has greatly improved people’s quality of life. At the Developer Conference in May 2016, Google announced that they would provide the Android Nought operating system with an interface to obtain GNSS raw observations, which is of epoch-making significance for smartphone positioning. In the early stages, few smartphones could collect high-quality GNSS raw observations. The Samsung Galaxy S7 was used to collect and analyze GNSS raw observations, and the results reflected that the quality of the smartphone antenna and its duty cycle would affect the observations [1]. Gim et al. [2] and Zhang et al. [3] analyzed the GNSS raw observations of the nexus9 tablet, incorporating the Doppler and carrier observations into the calculation. They concluded that the Carrier-to-Noise ratio (C/N0) of GNSS raw observations of mobile devices is 10 dB-Hz lower than the mean of the geodesic receiver, and the horizontal and vertical root mean square (RMS) errors reached 0.8 m and 1.4 m. After the Google Developer Conference, various manufacturers had successively launched smartphones that supported obtaining GNSS raw observations. Sikirica et al. [4] used the Huawei P10 smartphone to conduct the pseudorange point positioning test and the horizontal positioning accuracy could reach 10 m, but the positioning accuracy of elevation direction was poor. Specht et al. [5] used the Samsung Galaxy smartphones to conduct maritime differential positioning tests. The results showed that the horizontal positioning accuracy could also reach 10 m, which satisfied most marine navigation accuracy requirements. Although results obtained in the above experiments are of great significance to the improvement of the positioning accuracy of smartphones, there is still a certain gap between the positioning accuracy of geodetic receivers [6,7].

In May 2018, Xiaomi launched the first GNSS smartphone that supports (L1/E1+L5/E5) dual-frequency, which meant that smartphone positioning had entered a new stage [8]. Scholars began to study high-precision positioning algorithms based on smartphones [9,10]. Researchers [11,12] evaluated the GNSS raw observations of the Xiaomi 8 smartphone and found that the carrier was not affected by the duty cycle. Then, they conducted Real-Time Kinematic (RTK) and PPP tests and obtained horizontal RMS positioning errors of 1.17 m and 2.23 m. According to the phenomenon that the difference of the smartphone between the pseudorange observation value and the carrier is not fixed, Chen et al. [13] proposed a precise single-point positioning method. It is based on the dual clock difference of the smartphone. The horizontal and elevation positioning accuracy can reach 0.81 m and 1.65 m.

With the continuous improvement of smartphone performance, the quality of the GNSS raw observations of the smartphone has also improved. However, the ionospheric delay error still affects the positioning effect. Weakening the ionospheric delay is the key to improving the positioning accuracy of smartphones. The effect of Global Ionospheric Map (GIM) files to correct the ionospheric delay is significantly inferior to the post-GIM data [13], which is as difficult as the Klobuchar model to meet the real-time requirements of smartphone positioning. Liu et al. [14] proposed an ionospheric model based on spherical cap harmonic analysis, which accurately reflects regional TEC changes and provides ionospheric prediction functions. Rui et al. [15] used the Beidou and GPS dual systems to establish an ionospheric model in China. The results show that the dual systems can significantly improve the accuracy of the ionospheric model. A. Rovira-Garcia et al. [16] uses the observations obtained by CORS to monitor the changes in the inversion of regional or global ionospheric distribution in real-time. Researchers [17] presented an approach which used dual-frequency data from multiple low Earth orbit (LEO) satellites to generate a global topside ionospheric map, calculating the ionospheric electron content more accurately. Jon Bruno et al. [18] concluded that the smartphones showed potential as a useful course of TEC for ionospheric observations by analyzing the quality of smartphone-based TEC measurements. Ren et al. [19] proposed two approaches which can significantly improve the accuracy of the ionospheric model to combine GNSS and LEO observation data for ionosphere modeling.

At present, most ionospheric models use the method of segmented estimation. This method uses all epoch observations in a period to calculate a set of model parameters, which can obtain higher accuracy during the ionospheric quiet period. However, during the active period of the ionosphere, large errors will be caused by ignoring part of the local high-frequency information. Dual-frequency observations can monitor changes in the inversion area or global ionospheric distribution in real-time, but the amount of dual-frequency data on smartphones is small and the availability is low. Based on the above analysis, this article uses CORS dual-frequency GNSS observations to invert the ionospheric model, and transmit the results to the smartphone to achieve real-time ionospheric correction.

The experimental setup and raw observations analysis are described in Section 2. In Section 3, the positioning algorithm of smartphone is described in detail. The experimental results are presented in Section 4. The conclusion and discussion of this work are in Section 5.

## 2. Experimental Setup and Raw Observations Analysis

The equipment used in this experiment includes an OPPO Reno 10X Zoom smartphone, a Huawei P10 smartphone, two Xiaomi 8 smartphones, and a geodetic receiver (Hi-Target iRTK2). They all supported the acquisition of the GNSS raw observations from the four major satellite navigation systems: GPS, GLONASS, BDS, and GALILEO. To avoid the duty cycle affecting the quality of the experimental data, the four smartphones had the power-saving mode turned off. Among them, two Xiaomi 8 smartphones had different operating system versions; one system version was Android 8, which does not support mandatory tracking mode (which tracks all GNSS constellations and frequencies when the work cycle is disabled), and the other system version was For Android 9 (supported to enable mandatory tracking mode). The specific parameters of the experimental equipment are shown in Table 1.

**Figure 1:**
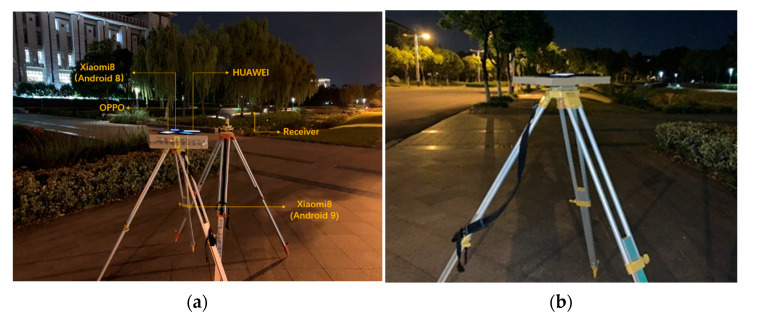
Schematic diagram of experimental. (**a**) Geodetic receivers and smartphones to obtain the GNSS raw observations (19 June 2020, 7 p.m.); (**b**) Positioning test on the smartphone (28 September 2020, 8 p.m.).

Two datasets were collected in the experiment, the sampling rate was set to 1 s, and the collection location was the north side of Li Wenzheng Library on the Jiulonghu Campus of Southeast University. The observation conditions of this site were good and it was in a low multipath environment. To ensure the accuracy and reliability of the experimental results, the network RTK technology was used to obtain the accurate coordinates of the control points before the experiment. The accuracy of the points can reach 2–3 cm in the horizontal and 5 cm in the elevation to meet the high-precision positioning requirements of smartphones. The first dataset was collected on 19 June 2020 and the observation time was 1 h. The equipment used was the above-mentioned four smartphones and a geodetic receiver (iRTK2), as shown in Figure 1a. This dataset is used for quality analysis of the GNSS raw observations. The second dataset was collected on 28 September 2020 and the observation time was 1 h. The device used was a Xiaomi 8 B smartphone, as shown in Figure 1b. This dataset was used to analyze the effect of the positioning experiment.

We developed the SEU_GNSSLog for data collecting, pre-processing and sending. In addition, a Windows desktop program was developed to invert the regional ionospheric model, and an APP was developed for real-time positioning.

To systematically analyze the quality of the GNSS raw observations of different smartphones, this article uses the experimental equipment mentioned in Table 1 to perform simultaneous observations in the same environment. Taking the observations of iRTK2 as a standard to analyze the number of visible satellites, signal continuity, the C/N0 of satellite, and pseudorange and carrier observations.

The Android system sets the satellite signals captured by the smartphone’s GNSS receiver to three levels: visible, synchronized, and tracking. Under normal circumstances, only at the traceable level, the GNSS raw observations output by the GNSS chip are accurate and credible.

**Figure 2:**
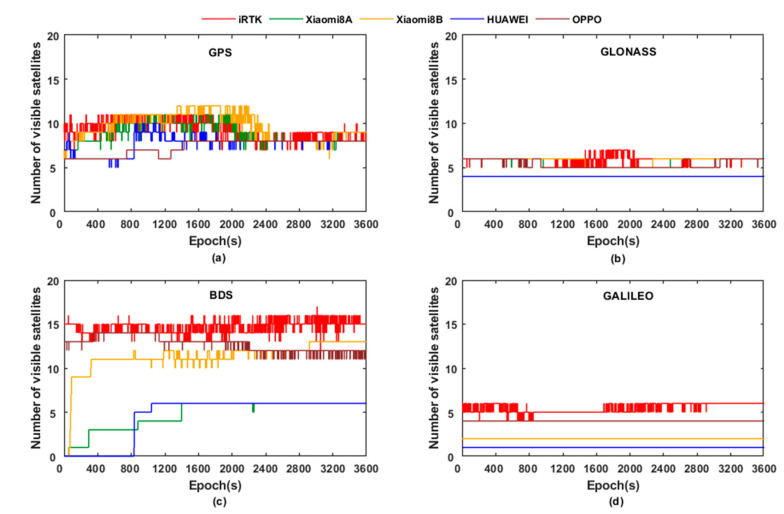
The number of visible satellites in each system observed by the experimental equipment. (**a**) The number of GPS observed by the experimental equipment; (**b**) The number of GLONASS observed by the experimental equipment; (**c**) The number of BDS observed by the experimental equipment; (**d**) The number of GALILEO observed by the experimental equipment.

Analyzing Figure 2a–d, it can be concluded that Xiaomi 8 B smartphone has a stronger ability to capture satellite signals than Xiaomi 8 A, this is because the Android 9 version is forced to enable full GNSS signal tracking so that the smartphone can track all GNSS constellations and frequencies when the work cycle is disabled. It can be shown that the number of satellites is greater than 10, which can meet the positioning needs, but there is still a certain gap compared with the geodetic receiver. For GPS and GLONASS, the number of satellites observed by the smartphones and geodetic receivers is similar; for BDS, the number of BDS satellites that can be observed by geodetic receivers is about 15, which reflects the fact that the BeiDou Navigation Satellite System has good coverage over our country’s territory.

**Figure 3:**
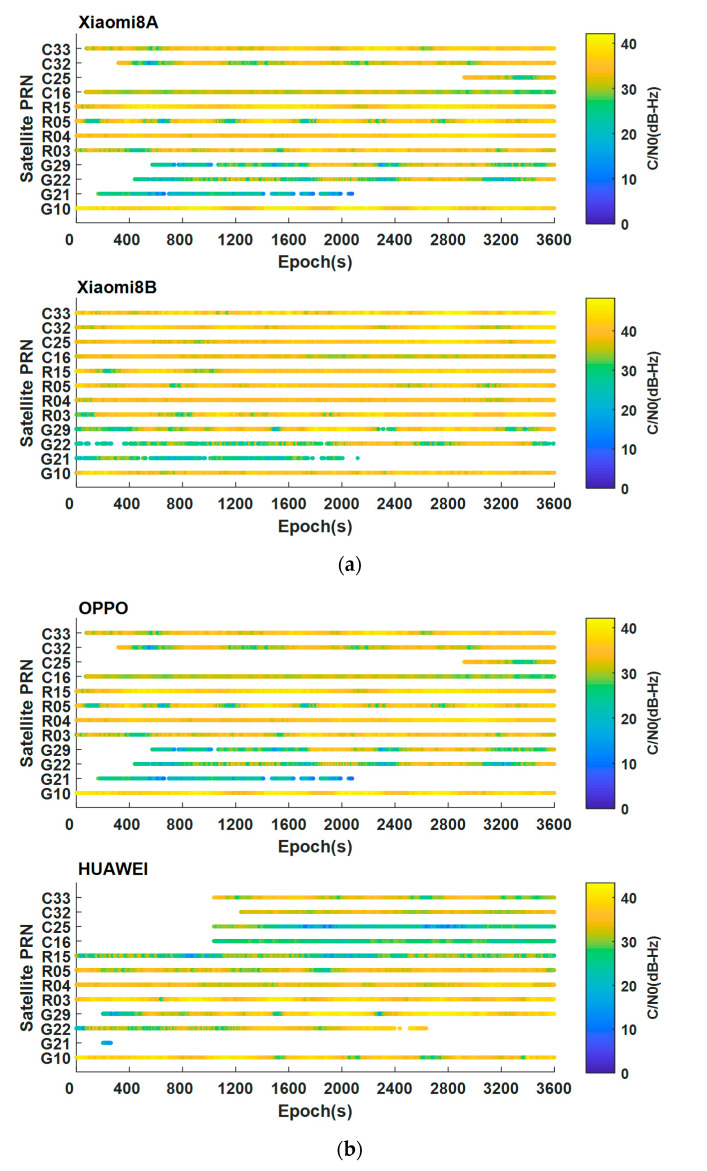

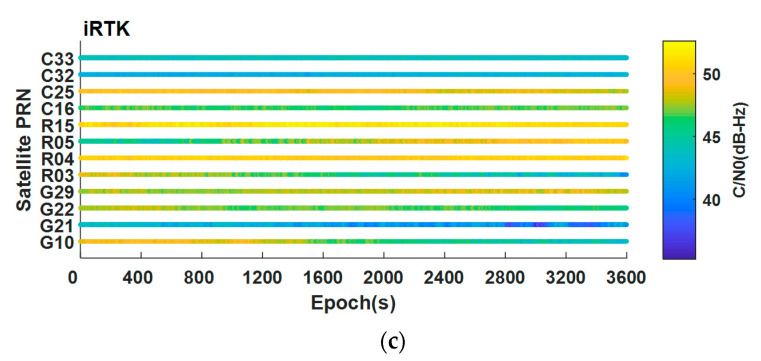
Continuity and the C/N0 of the GNSS raw observations from smartphones and receivers. (**a**) Continuity and the C/N0 of Xiaomi 8 A and Xiaomi 8 B; (**b**) Continuity and the C/N0 of OPPO and HUAWEI; (**c**) Continuity and the C/N0 of iRTK2.

As the number of GALILEO satellites captured by each smartphone is small, this article selects GPS, GLONASS, and BDS system satellites to analyze the continuity and the C/N0 of the GNSS raw observations. Analyzing Figure 3a, it can be seen that the continuity and signal strength of the Xiaomi 8 B with forced tracking is significantly better than those of Xiaomi 8 A, and the satellite signal capture capability is stronger. Taking the G21 satellite as an example, it can be found that all smartphones have a signal loss-of-lock phenomenon, but the moments of losing lock are different, and satellite signals will be reacquired at certain times. Xiaomi 8 B and OPPO smartphones have a stronger ability to track BDS satellite signals than Xiaomi 8 A and HUAWEI smartphones, but the quality of observations collected by Xiaomi 8 B is better.

To further analyze the availability of GNSS raw observations of smartphones, this article compares the pseudorange and carrier observations. Due to the pseudorange and carrier of smartphones use different clocks [13], we deal with them as follows: Firstly, a reference satellite is selected separately in each system, then the difference between reference satellite and others is calculated to obtain the combined observations to eliminate the influence of the equipment clock error; secondly, calculating the difference between the epochs of the obtained combined observations to obtain its rate of change; finally, taking the results of iRTK as the standard, the root mean square error (RMSE) of each smartphone is calculated.

**Figure 4:**
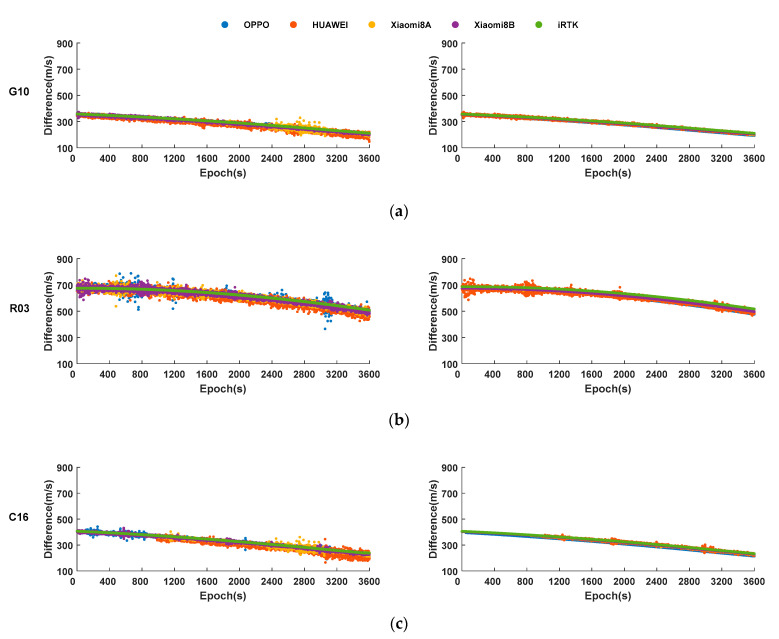
The rate of change of pseudorange and carrier obtained by smartphones and iRTK2 after processing. (**a**) The rate of change of pseudorange and carrier of G10. (**b**) The rate of change of pseudorange and carrier of R03. (**c**) The rate of change of pseudorange and carrier of C16.

Figure 4 shows the rate of change of the pseudorange and carrier combinative observation processed by each experimental device. Due to space limitations, each satellite system selects only one for display (the Galileo satellite observations are not be considered because the amount of it is small). It can be found from Figure 4 that the change rate of the combined pseudorange and carrier observations of each satellite is consistent with the results of the geodetic receiver in terms of numerical value and change law, while in stability, there is still a certain gap compared with geodetic receivers. Comparing the satellites of different systems in Table 2, it is found that most RMSE of pseudorange of the GPS and BDS are less than 15 m/s, and the RMSE of carrier are less than 10 m/s. The data accuracy is significantly better than that of the GLONASS.

Strikingly, the experiments found that some satellites’ carrier combinative observations have gross errors or systematic errors for which the cause is not clear, and the influence of these errors is greater than 1000 m/s. In addition, the RMSE of Xiaomi 8 A and Xiaomi 8 B is significantly smaller than that of HUAWEI and OPPO smartphones. Among them, Xiaomi 8 B has the best data accuracy, which is consistent with the conclusion that Xiaomi 8 B can effectively improve the quality of the GNSS raw observations of the smartphone after enabling the mandatory tracking model.

Based on the above analysis, this article uses Xiaomi 8 B smartphones for subsequent positioning experiments.

## 3. Positioning Algorithm of Smartphone

### 3.1. Regional Ionospheric Correction Model

How to accurately extract the TEC value is the basis for inverting the ionospheric correction model using regional CORS observations. In this article, the carrier-smoothing pseudorange method is used to extract TEC. This method not only has high accuracy but also meets the real-time requirements of smartphones. The TEC value can be expressed as:

(1) $$TEC=\frac{f_{1}^{2}f_{2}^{2}}{40.28\left(f_{1}^{2}-f_{2}^{2}\right)}\left(\bar{P_{d}}+B^{s}+B_{r}\right)$$

where $B^{s}$ is the hardware delay on the satellite, $B_{r}$ is the hardware delay on the receiver, $f_{1},f_{2}$ is the first frequency and second frequency of a dual-frequency signal, ${\bar{P}}_{d}$ is the smoothed dual-frequency pseudorange observations, ${\bar{P}}_{d}=P_{2}-P_{1}$, $P_{1},P_{2}$ is the first frequency and second frequency of the pseudorange observations. The smoothing formula is as follows:

(2) $${\bar{P}}_{k}=a_{k}P_{k}+\left(1-a_{k}\right)\left({\bar{P}}_{k-1}+L_{k}-L_{k-1}\right)$$

where the subscript k is observation epoch, L is the dual-frequency carrier observation; a is smoothing weight, the initial value of it is 1 and it decreases as the epoch increases. After being smaller than a certain threshold, it remains unchanged.

Through GNSS observations, only the TEC on the signal propagation path can be extracted. In practical applications, the known points and the measured points are often far away. Therefore, it is necessary to extend the discrete data to the entire measurement area through a mathematical model. In this article, the spherical harmonics (SH) model is used to simulate the ionospheric distribution. The SH function can be expressed as:

(3) $$VTEC=\sum_{n=0}^{n_{\max}}\sum_{m=0}^{n}{\tilde{C}}_{nm}\left(\sin\varphi_{IPP}^{\prime }\right)\left(A_{nm}\cos m\theta +B_{nm}\sin m\theta \right)$$

where VTEC is TEC in the vertical direction, $n_{\max}$ is the highest order of expansion, m is the number of expansions, ${\tilde{C}}_{nm}$ is the fully normalized Legendre function, $\varphi_{IPP}^{\prime }$ is the geocentric latitude of the pierce point, θ is the daily fixation accuracy of pierce point, and $A_{nm},B_{nm}$ is the function model parameters.

At present, the commonly used ionospheric file uses the 15-order spherical harmonic function model with a time interval of 2 h and 256 parameters to be estimated. For a small area, the parameters to be estimated can be reduced to 6, and the simplified equation can be expressed as:

(4) $$\begin{array}{l} VTEC=C_{0,0}\left(\sin\varphi_{IPP}^{\prime }\right)A_{0,0}+C_{1,0}\left(\sin\varphi_{IPP}^{\prime }\right)A_{1,0}\\ \;\;\;\;\;\;\;\;\;\;\;\;\;+C_{1,1}\left(\sin\varphi_{IPP}^{\prime }\right)\left(A_{1,1}\cos\theta +B_{1,1}\sin\theta \right)\\ \;\;\;\;\;\;\;\;\;\;\;\;\;+C_{2,2}\left(\sin\varphi_{IPP}^{\prime }\right)\left(A_{2,2}\cos2\theta +B_{2,2}\sin2\theta \right) \end{array}$$

where $C_{n,0}\left(n=1,2,3,4\right)$ is the Legendre polynomials, $A_{0,0}$,$A_{1,0}$, $A_{1,1}$, $B_{1,1}$, $A_{2,2}$, $B_{2,2}$ are model parameters.

According to Formula (3), the use of low-order SH functions to fit the regional ionospheric TEC can better describe the spatial distribution and change of TEC. Combining the Formula (1) to obtain the observation equation:

(5) $${\bar{P}}_{d}=40.28MF\frac{f_{1}^{2}-f_{2}^{2}}{f_{1}^{2}f_{2}^{2}}\sum_{n=0}^{n_{\max}}\sum_{m=0}^{n}{\tilde{C}}_{nm}\left(\sin\varphi_{IPP}^{\prime }\right)\left(A_{nm}\cos m\theta +B_{nm}\sin m\theta \right)-B_{r}-B^{s}$$

where MF is the single-layer projection function, the meaning of the rest of the parameters is the same as above.

In this article, the Kalman filter is used to estimate real-time ionospheric model parameters. Assuming that the number of single-epoch CORS stations is n, the number of observable satellites is m, the state vector X and the observation vector Z are expressed as:

(6) $$\begin{array}{l} X={\left[a_{1},\cdots ,a_{6},B_{1},\cdots ,B_{n},B^{1},\cdots ,B^{m}\right]}^{T}\\ Z={\left[{\bar{P}}_{1},\cdots ,{\bar{P}}_{mn}\right]}^{T} \end{array}$$

where $\left[a_{1},\cdots ,a_{6}\right]$ are the model parameters, $\left[B_{1},\cdots ,B_{n}\right]$ are the hardware delay of CORS station, $\left[B^{1},\cdots ,B^{m}\right]$ are the hardware delay of the satellite. The hardware delays of satellites and receivers are linearly correlated, leading to a rank defect in the design matrix. The zero-mean benchmark is usually added for constraint. It is assumed that the sum of the hardware delays of the satellites in each system is 0. Since only part of the satellites can be observed by regional CORS, this paper adds the zero-mean constraint of receiver hardware delay, which can be expressed as $\sum_{i=1}^{n}B_{i}=0$. This method uses different strategies to separate the hardware delays of satellite and receiver, resulting in a systematic deviation between the obtained satellite hardware delay and the DCB product, but it will not affect the calculation of the ionospheric model parameters.

### 3.2. The Point Positioning Model of Smartphone

GNSS point positioning does not require reference station data and is easy to implement. It is the main application method of smartphone GNSS positioning. This article uses carrier-smoothing pseudorange point positioning and precise point positioning to verify the model effect. The carrier-smoothing formula can be expressed as:

(7) $$\begin{array}{l} \bar{P}\left(t_{1}\right)=P\left(t_{1}\right)\\ \bar{P}\left(t_{i}\right)=\frac{1}{i}\cdot P\left(t_{i}\right)+\frac{i-1}{i}\cdot \left[\bar{P}\left(t_{i-1}\right)+\varphi \left(t_{i}\right)-\varphi \left(t_{i-1}\right)\right] \end{array}$$

where P is the pseudorange, $\bar{P}$ is the pseudorange after smoothing, $t_{i}$ is the epoch, φ is the carrier. However, for Android smartphones, the difference between pseudorange and the carrier is not fixed. The increments of the pseudorange between adjacent epochs and the carrier are not equal:

(8) $$\varphi \left(t_{i}\right)-\varphi \left(t_{i-1}\right)=P\left(t_{i}\right)-P\left(t_{i-1}\right)+d_{i}$$

where d is the difference between carrier increment and pseudorange increment. When Formula (7) is used for calculation, the pseudorange obtained will contain the cumulative incremental error. To solve the impact of it on the single point positioning of the carrier-smoothing pseudorange, this article uses a fixed smoothing window (the size of window is 30) to ensure that all pseudorangein the same epoch are smoothed the same number of times, so that all pseudorange have the same cumulative incremental error. This error can be absorbed by the device clock error during the Kalman filtering process and does not affect the positioning result.

According to the uncertainty of the difference between the GNSS pseudorange and the carrier in the smartphone, it is considered that they have different device clock parameters.

Currently, the availability of dual-frequency GNSS raw observations of smartphones is poor. In this article, only single-frequency GNSS data is used for PPP positioning, and the Kalman filter is used for parameter estimation. The observation equations of the pseudorange and the carrier are:

(9) $$\begin{array}{l} P=\rho +c\cdot d{\tilde{t}}_{P}-c\cdot dT+d_{trop}+d_{orb}+d_{ion}+\varepsilon \\ \varphi =\rho +c\cdot d{\tilde{t}}_{\varphi }-c\cdot dT+d_{trop}+d_{orb}+d_{ion}+\tilde{N}+\varepsilon \end{array}$$

where ρ is the geometric distance between satellite and receiver, $d{\tilde{t}}_{P},d{\tilde{t}}_{\varphi }$ are the clock error of pseudorange and the carrier, dT is the clock error of the satellite, $\tilde{N}$ is the ambiguity of whole cycles, $d_{trop}$ is the tropospheric delay, $d_{orb}$ is the error of satellite ephemeris, $d_{ion}$ is the ionospheric delay, ε is the residual. Since cycle slips data accounted for more than 20% of the smartphone phase observations and cycle slips occur frequently [20,21], this article does not fix the carrier phase.

## 4. Results

**Figure 5:**
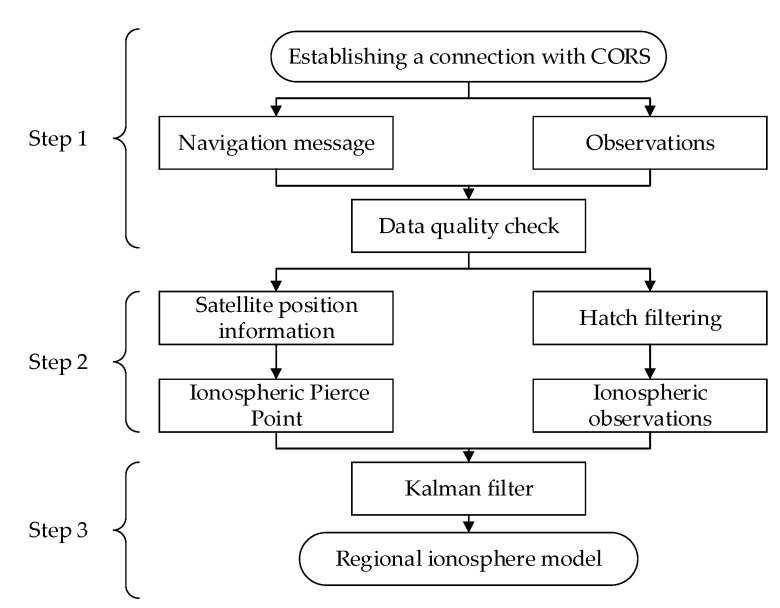
The flow chart of real-time regional ionospheric model.

According to the principle described in Section 4, the regional ionospheric delay model is established by using the real-time observations of four CORS in Nanjing area, and the update interval of the model is 5 s. Smartphone obtains the model data in real-time for positioning experiments. The real-time ionospheric modeling process is as shown in Figure 5 and the descriptions of steps are as follows:

1. Receive real-time dual-frequency observations and navigation messages from CORS, and perform data preprocessing;
2. Smooth the ionospheric observations and calculate the latitude and longitude of the pierce point;
3. The observation vector of all CORS in a single-epoch is used to form an observation vector to construct an observation equation; the Kalman filter is used to filter the parameters in real-time to obtain a regional ionospheric model.

**Figure 6:**
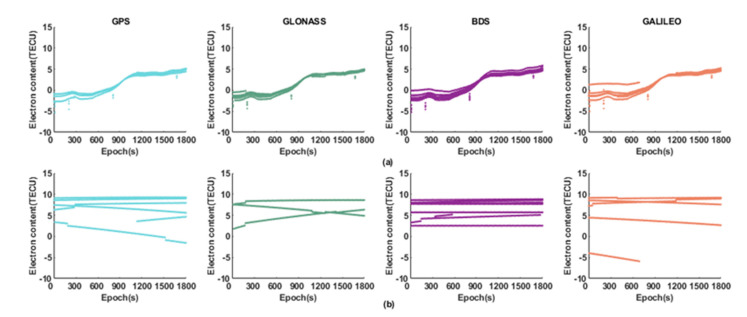
Electron content error at the pierce point. (**a**) Regional ionospheric model; (**b**) Klobuchar model.

**Figure 7:**
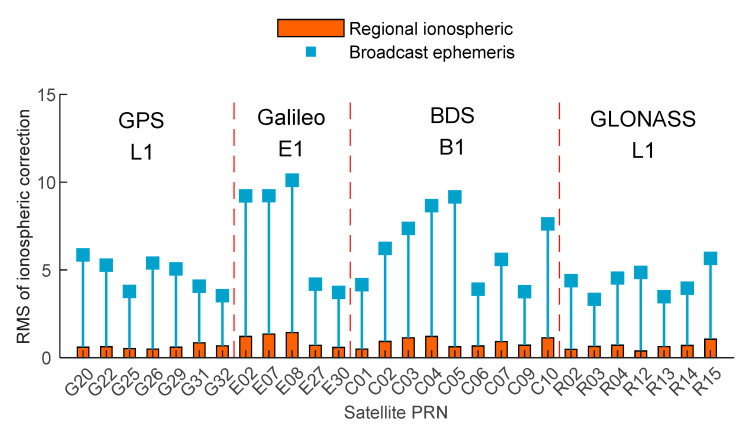
The RMS of ionospheric delay correction of the Klobuchar and the regional ionospheric model.

GIM products have good applicability, and the post-processing GIM data can eliminate 80% of the ionospheric delay error. Therefore, this article regards the post-processing GIM file published by CODE as a standard. The Klobuchar model and the regional ionospheric model are used to calculate the ionospheric delay and electron content at the pierce point, and compare them with the standard to calculate the residuals of each satellite, as shown in Figure 6 and Figure 7.

As shown in Figure 7, the results of the Klobuchar remain basically unchanged during the experiment, and the results of the regional ionospheric model are changed with time slowly. In addition, the accuracy of the regional ionospheric model is less than 5 TECU for each system, and the error of the electron content calculated by the Klobuchar model is 5–10 TECU. It can be concluded that the regional ionospheric model can accurately reflect the ionospheric TEC distribution and its changes by comparing the results of the two models.

Since the dual-frequency GNSS observations received by the smartphone are fewer and the quality is poor, the single-frequency data is selected for the ionospheric delay analysis. It can be seen from Figure 7 that the ionospheric correction error calculated by the Klobuchar model is about 5 m, and some satellites reach 10 m, while the error calculated by the regional ionospheric correction model is maintained at about 1 m. It can be found the regional ionospheric model can calculate the ionospheric delay correction more accurately.

Based on the above analysis, it can be considered that the regional ionospheric model has higher accuracy than the Klobuchar model. To verify the influence of the regional ionospheric model on the positioning results of smartphones, this article uses Xiaomi 8 B smartphone for positioning experiments, and selects carrier-smoothing pseudorange and PPP positioning model. The experimental plan is as follows:

1. Ignore the ionospheric error and make no corrections;
2. Download the broadcast ephemeris and use Klobuchar to calculate the ionospheric delay;
3. Use observations of CORS to invert the regional ionospheric correction model to calculate the ionospheric delay in real-time.

**Figure 8:**
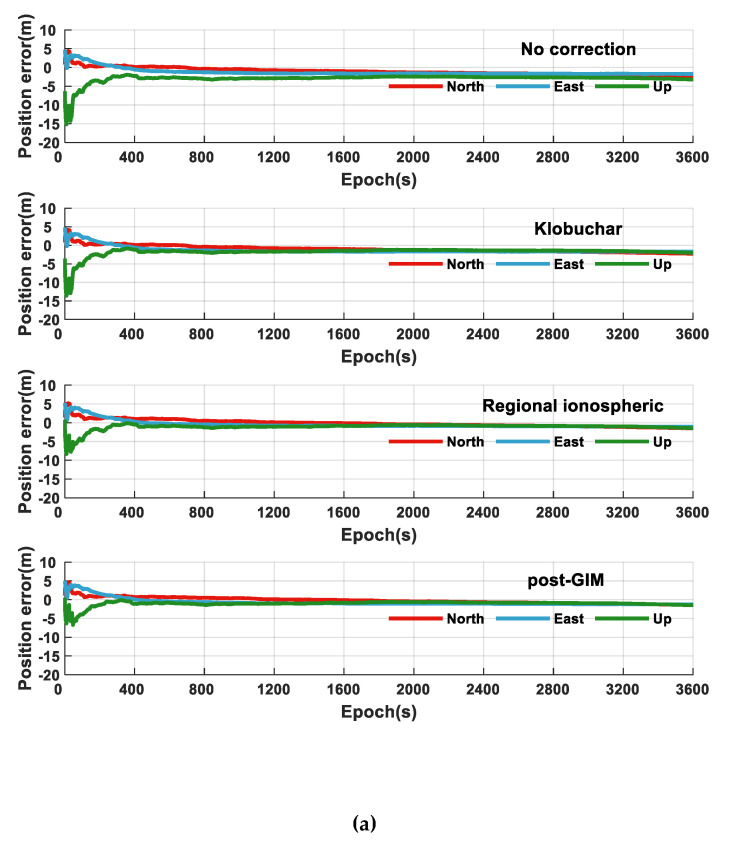

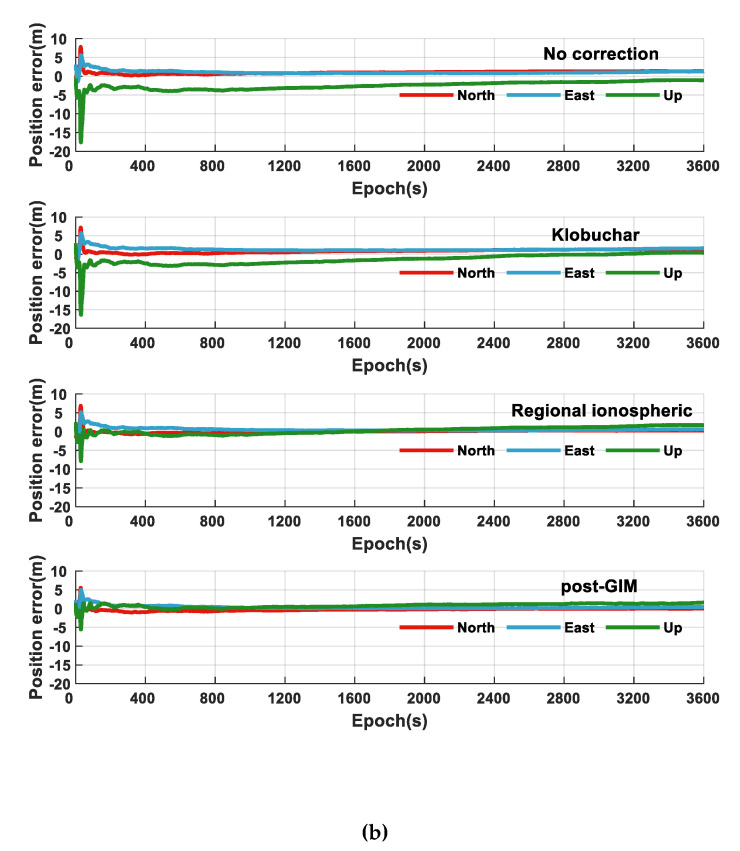
The positioning results of the Xiaomi 8 B smartphone. (**a**) Carrier-smoothing pseudorange positioning, (**b**) PPP (the no correction means no ionospheric correction).

It can be seen from Figure 7 and Figure 8 and Table 3 that the ionospheric delay error is one of the errors that cannot be ignored in the process of high-precision positioning of smartphones. Ignoring the influence of the ionosphere, the accuracy of pseudorange positioning results is low, the horizontal can reach 4 m, and the elevation can reach 10 m. It needs be weakened by appropriate models. Analyzing the influence of Klobuchar and regional ionospheric models on the results of the two positioning models, this article finds that using either pseudorange positioning or carrier positioning, the regional ionospheric model can effectively improve the positioning accuracy of smartphones. Additionally, the positioning accuracy of the regional ionospheric model is similar to the post-GIM products. Among them, using the regional ionospheric model for PPP positioning can achieve a positioning accuracy of 1 m.

To evaluate the influence of the regional correction model on the convergence time of smartphone single-frequency PPP positioning, this article sets up five experiments, each of them taking 10 min. The convergence condition is that the range of continuous 20 s is less than 0.5 m. It can be seen from Figure 8b that the ionospheric correction mainly affects the accuracy of the elevation direction; this article does not analyze the horizontal convergence time. What stands out in the Table 4 is that the regional correction model can shorten the convergence time in the elevation direction compared with the Klobuchar model, which proves that the regional ionospheric model can more accurately correct the ionospheric delay error of smartphones. It is of great significance for the high-precision positioning of smartphones.

## 5. Conclusions and Discussion

In this article, we have compared and analyzed the quality of the GNSS raw observations of different smartphones, and adopted the regional ionospheric correction model to improve the positioning accuracy of the smartphone.

Taking the observations of the geodetic receiver as the standard value, analyzing the GNSS raw observations of OPPO Reno 10X Zoom, Huawei P10, Xiaomi 8 A and B smartphones, we found that the ability of smartphones to acquire GPS and GLONASS satellite signals is similar to that of geodetic receivers. For the BDS, the number of satellites that can be observed by the geodetic receiver is about 15, and the performance of each smartphone is different. Among them, the Xiaomi 8 B smartphone performs best, and the number of BDS that can be observed is stable at about 10. There is a positive correlation between the C/N0 and the continuity of the observations, but a very obvious signal loss-of-lock phenomenon occurs in each smartphone during the observation experiment. Through further analysis of the pseudorange and carrier, it can be concluded that the change rate of the combined pseudorange and carrier observations of each satellite is consistent with the results of the geodetic receiver in terms of numerical value and change law while, in stability, there is still a certain gap compared with geodetic receivers.

The GIM file is used as the standard to interpolate the electron content of the pierce point and the ionospheric delay correction, comparing the results of the Klobuchar model and the regional ionospheric correction model with this standard. During the experiment, the electron content calculated by the regional ionospheric model changes slowly with time, and the accuracy of each system is less than 5TECU. It can more accurately reflect the changes of the ionosphere than Klobuchar. In addition, the accuracy of the ionospheric delay correction calculated by the regional ionospheric model is up to 1 m. To further study the influence of the regional ionospheric model on positioning, this article performs carrier-smoothing pseudorange and single-frequency PPP positioning. Compared with the Klobuchar, the positioning accuracy of the regional model is improved, especially in the elevation direction. The accuracy of the regional ionospheric model is similar to the post-GIM product, which means that the regional model has a significant effect on ionospheric correction. Meanwhile, the use of the regional ionospheric model can shorten the convergence time of the elevation direction to less than 1 min, which can greatly improve the positioning performance of smartphones.

Previous studies have found that the data quality of GPS and Galileo L5/E5 frequency is better than L1/E1 [22,23,24], and the use of L1/E1 and L5/E5 dual-frequency data can effectively weaken ionospheric delay. However, due to the fact that the number of dual-frequency data obtained by the smartphone is small, the utilization rate of the dual-frequency data is low. How to effectively use the dual-frequency data is the focus of the next research. During the experiment, we found that the carrier of the smartphone fluctuates greatly, and the lack of carrier often occurs, resulting in low carrier data utilization. After analysis, it can be seen that the data characteristics of Doppler observations are similar to those of carrier observations. Whether the Doppler data can replace the carrier to complete positioning appropriately when the smartphone cannot output the carrier, or whether the carrier has problems, are also the focus of future research. In addition, the antenna also affects the improvement of positioning performance [25] and we can try to establish a suitable model to correct it in the next research.

## Figures and Tables

**Table 1:** Equipment related parameters and experimental settings.

Device	Version	Sampling Rate	Observation Time	Global System
OPPO Reno *10*X Zoom	Android *9*	*1* Hz	*1* h	GPS/GLO/GAL/BDS/QZS
HUAWEI P*10*	Android *9*	*1* Hz	*1* h	GPS/GLO/GAL/BDS
Xiaomi *8* A	Android *8*	*1* Hz	*1* h	GPS/GLO/GAL/BDS
Xiaomi *8* B	Android *9*	*1* Hz	*1* h	GPS/GLO/GAL/BDS
Hi-Target iRTK*2*		*1* Hz	*1* h	GPS/GLO/GAL/BDS/QZS

**Table 2:** The RMSE of combinative observation of each smartphone (Based on the results of iRTK2).

	OPPO		HUAWEI		Xiaomi 8 A		Xiaomi 8 B	
	Pseudorange	Carrier	Pseudorange	Carrier	Pseudorange	Carrier	Pseudorange	Carrier
G*10*	10.042	4.562	6.712	2.582	4.601	1.151	3.915	1.108
G*12*	22.681	12.047	17.241	9.184	12.021	6.848	5.064	3.395
G*21*	15.746	8.174	11.203	5.723	7.497	3.486	4.138	3.069
G*29*	9.845	5.741	8.607	3.363	5.452	2.105	4.913	1.947
R*03*	19.835	12.765	17.754	8.306	13.333	3.646	12.984	3.620
R*04*	24.629	13.451	19.328	11.136	16.763	8.667	14.029	8.019
R*05*	11.284	9.563	10.416	8.184	9.796	7.756	8.910	6.924
R*15*	15.206	10.843	14.094	9.356	12.166	8.661	10.549	7.940
C*16*	13.219	10.712	7.652	4.318	6.111	2.887	5.092	2.193
C*25*	11.271	9.007	8.492	6.477	5.317	3.371	3.749	3.065
C*32*	16.816	11.054	12.592	9.710	8.476	6.455	6.013	5.201
C*33*	10.480	8.183	9.705	7.659	6.429	3.622	5.481	2.872

**Table 3:** RMSE of the positioning result.

Method	Model	Horizontal (m)	Elevation (m)	Promotion (Horizontal)	Promotion (Elevation)
Carrier-smoothing pseudorange	No correction	3.364	3.212		
	Klobuchar	2.758	2.586	18%	20%
	Regional	2.107	1.687	38%	48%
	Post-GIM	2.004	1.425	40%	56%
PPP	No correction	2.427	2.698		
	Klobuchar	1.708	1.874	30%	31%
	Regional	0.836	1.081	65%	60%
	Post-GIM	0.753	0.917	69%	66%

**Table 4:** Convergence time in elevation direction of PPP.

	Klobuchar (s)	Regional (s)
*1*st	94	59
*2*nd	99	42
*3*rd	83	47
*4*th	88	52
*5*th	91	45

## Data Availability

Data sharing is not applicable to this article.
